# Cap-binding complex (CBC)

**DOI:** 10.1042/BJ20131214

**Published:** 2013-12-20

**Authors:** Thomas Gonatopoulos-Pournatzis, Victoria H. Cowling

**Affiliations:** *MRC Protein Phosphorylation Unit, College of Life Sciences, University of Dundee, Dow Street, Dundee DD1 5EH, U.K.

**Keywords:** cap-binding complex (CBC), 7-methylguanosine cap (7mG), splicing, transcription, translation, ABA, abscisic acid, Ars2, arsenite-resistance protein 2, CBC, cap-binding complex, Cbp, cap-binding protein, Cdk9, cyclin-dependent kinase 9, CF IA, cleavage factor IA, CTD, C-terminal domain, CTIF, CBC-dependent translation initiation factor, DSIF, DRB sensitivity-inducing factor, eIF, eukaryotic initiation factor, hnRNP, heterogeneous nuclear ribonucleoprotein, MIF4G, middle domain of eIF4G, 7mG, 7-methylguanosine cap, mRNP, messenger ribonucleoprotein, mTORC1, mammalian target of rapamycin complex 1, NELF, negative elongation factor, NMD, nonsense-mediated decay, PABP, poly(A)-binding protein, PARN, poly(A)-specific ribonuclease, PHAX, phosphorylated adaptor of RNA export, PTC, premature termination codon, P-TEFb, positive transcription elongation factor b, RAM, RNMT-activating mini-protein, RNA pol II, RNA polymerase II, RNMT, RNA (guanine-7-)methyltransferase, RRM, RNA-recognition motif, snRNP, small nuclear ribonucleoprotein, SRSF1, serine/arginine-rich splicing factor 1, TMG, 2,2,7-trimethylguanosine cap, TREX, transcription export complex

## Abstract

The 7mG (7-methylguanosine cap) formed on mRNA is fundamental to eukaryotic gene expression. Protein complexes recruited to 7mG mediate key processing events throughout the lifetime of the transcript. One of the most important mediators of 7mG functions is CBC (cap-binding complex). CBC has a key role in several gene expression mechanisms, including transcription, splicing, transcript export and translation. Gene expression can be regulated by signalling pathways which influence CBC function. The aim of the present review is to discuss the mechanisms by which CBC mediates and co-ordinates multiple gene expression events.

## INTRODUCTION

In the eukaryotic cell, the maturation and translation of RNA pol II (RNA polymerase II) transcripts into proteins requires a co-ordinated series of effective and efficient processing events. The first mRNA processing event is the formation of the 7mG (7-methylguanosine cap) at the 5′ end of nascent transcripts. Subsequent processing and translation is largely dependent on this structure. Protein complexes including CBC (cap-binding complex) and the eIF4F (eukaryotic initiation factor 4F) bind to 7mG and recruit the enzymes and factors to the transcript which mediate further processing, export and translation.

## 7mG SYNTHESIS

**Figure 1:**
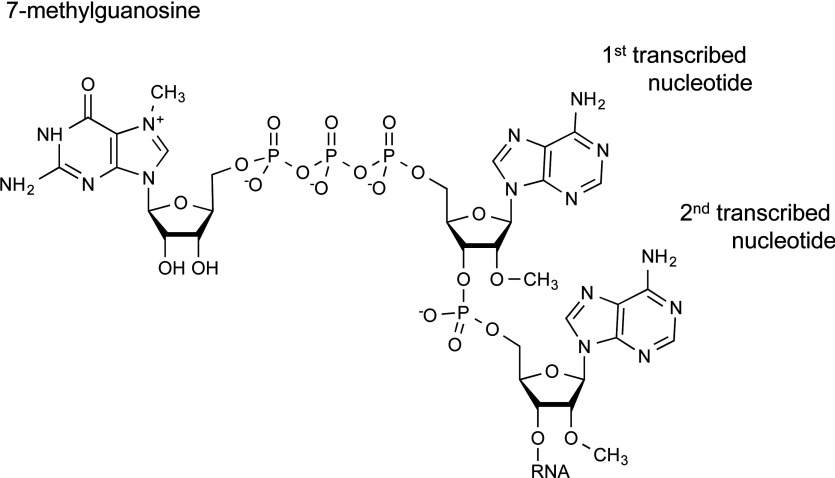
7mG structure The 7mG structure is depicted including the first and second transcribed nucleotides. Cap 2 structure is depicted, i.e. methylated on the O-2 position of ribose on the first and second transcribed nucleotides.

The first transcribed nucleotide of RNA pol II transcripts is modified by the addition of 7-methylguanosine during the early stages of transcription (Figure 1). All transcripts are synthesized with a 5′ triphosphate on the first nucleotide to which 7-methylguanosine is joined via a 5′–5′ triphosphate bridge, creating 7mG(5′)ppp(5′)X (X is the first nucleotide). Throughout the present review we use the abbreviation 7mG to refer to 7-methylguanosine in the cap structure. The 5′–5′ triphosphate linkage that joins 7-methylguanosine to the first nucleotide is thought to be found uniquely on RNA pol II transcripts. This unique structure enables certain processing factors to be recruited exclusively to RNA pol II transcripts [1–3]. Three enzymatic activities catalyse the addition of 7-methylguanosine: triphosphatase, guanylyltransferase and methyltransferase. The triphosphatase cleaves the terminal phosphate of the transcript and an RNA guanylyltransferase catalyses the addition of guanosine monophosphate to create G(5′)ppp(5′)X. Subsequently, a guanine-7-methyltransferase catalyses the addition of the methyl group to the N-7 position of the guanosine cap, creating 7mG(5′)ppp(5′)X [2,4,5]. In lower eukaryotes these three enzymatic activities reside in three distinct polypeptides; however, in metazoans the triphosphatase and guanylyltransferase reside in the same polypeptide, RNGTT [6]. In addition, in vertebrates the cap methyltransferase is a complex of two proteins; the catalytic subunit, RNMT [RNA (guanine-7-)methyltransferase], and the activating subunit, RAM (RNMT-activating mini-protein) [7].

**Figure 2:**
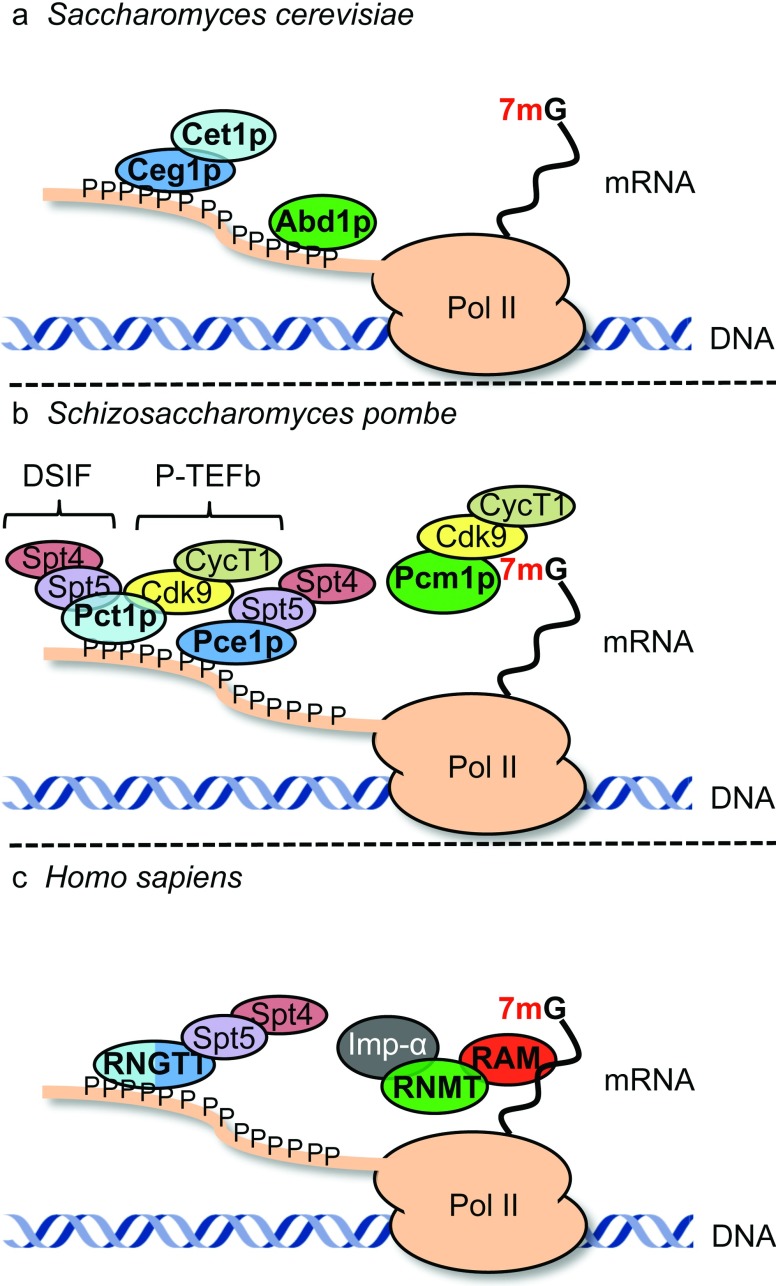
7mG synthesis in *S. cerevisiae*, *Schizosaccharomyces pombe* and humans The RNA triphosphatase enzymes are depicted in light blue, the RNA guanylyltransferase in dark blue and the RNA guanine-7-methyltransferases in green. (**a**) In *S. cerevisiae*, the RNA guanylyltransferase (Ceg1p) and the RNA triphosphatase (Cet1p) interact with the Ser^5^-phosphorylated RNA pol II CTD via the Ceg1p subunit [168–172]. The RNA guanine-7-methyltransferase (Abd1p) is recruited to the transcription initiation site via an interaction with phosphorylated RNA pol II CTD [12,95,173]. (**b**) In *S. pombe*, the RNA triphosphatase (Pct1p) and the RNA guanylyltransferase (Pce1p) independently interact with phosphorylated RNA pol II CTD [171,174]. Pct1p interacts with DSIF (DRB sensitivity-inducing factor) (Spt4/Spt5) and P-TEFb (Cdk9/Cyclin T1) [175,176]. Pce1p interacts with DSIF [176,177]. The RNA guanine-7-methyltransferase (Pcm1p) functions at the transcription initiation site, but does not physically associate with RNA pol II. Pcm1p and P-TEFb are in a complex and Pcm1p is required for P-TEFb recruitment to chromatin and transcription elongation [11,178,179]. (**c**) In humans, the RNA triphosphatase and the RNA guanylyltransferase activities reside in the same polypeptide, RNGTT. RNGTT interacts with DSIF (Spt4/5), which stimulates its activity up to 5-fold [13,180]. RNGTT promotes transcription elongation independently of its catalytic activity by overcoming NELF-dependent transcriptional pausing [13]. RNGTT is recruited to transcription initiation sites via an interaction with Ser^5^-phosphorylated RNA pol II CTD, which stimulates guanylyltransferase activity [169,181,182]. The RNA guanine-7-methyltransferase complex (RNMT/RAM) interacts with RNGTT and indirectly with RNA pol II [5,183]. RNMT also interacts with importin-α (Imp-α), which stimulates cap methyltransferase activity [184].

7mG is exclusively added to transcripts synthesized by RNA pol II since only this polymerase has a CTD (C-terminal domain), which when phosphorylated during the initial stages of transcription recruits the capping enzymes [8–10] (Figure 2). The capping enzymes have been demonstrated to promote transcription in lower and higher eukaryotes independently of their catalytic activities [11–13]. Although capping occurs predominantly during transcription, accumulating evidence suggests that cap-like structures can be also formed post-transcriptionally [14].

**Figure 3:**
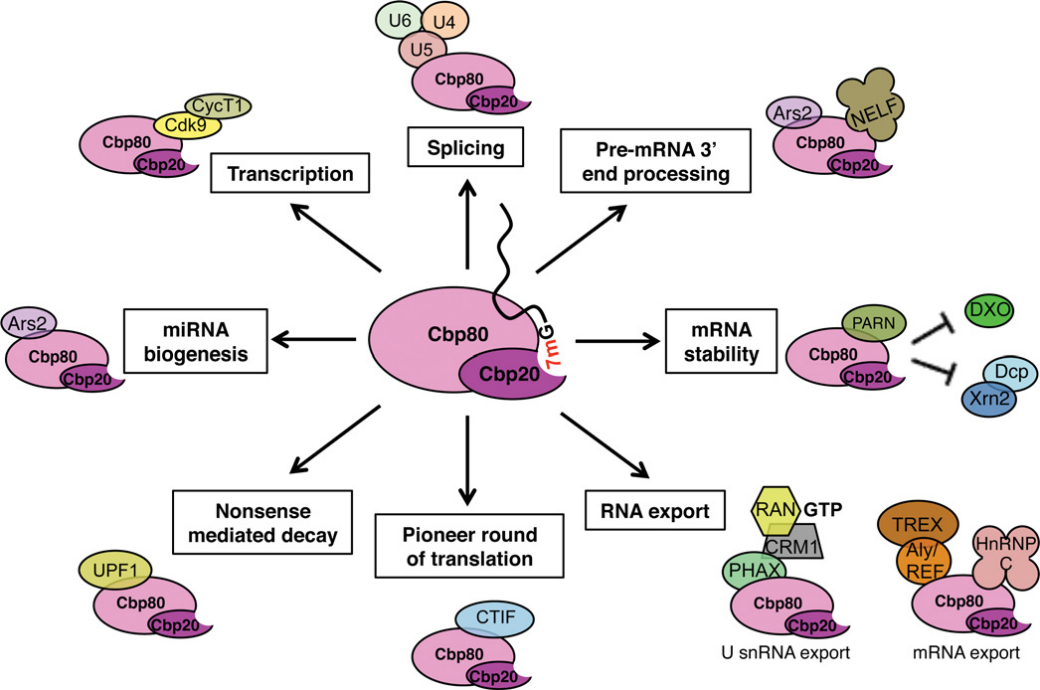
CBC functions CBC, composed of Cbp20 and Cbp80 subunits, binds to 7mG located at the 5′ end of RNA pol II transcripts. CBC interacts with a spectrum of factors mediating RNA metabolism and translation mechanisms. CycT1, cyclin T1; Dcp, decapping mRNA; DXO, decapping exoribonuclease; Xm2, 5′–3′ exoribonuclease 2.

The cap structure protects transcripts from exoribonucleolytic degradation [15,16]. Furthermore, it interacts with nuclear and cytoplasmic cap-binding proteins which mediate additional 7mG functions (Table 1 and Figure 3). CBC and eIF4F are the most thoroughly characterized cap-binding complexes, although other cap-binding proteins have been reported, including PARN [poly(A)-specific ribonuclease] deadenylase, PABPC1 [poly(A)-binding protein C1], PUM2 and Y14/Magoh [17–23].

**Table 1:** CBC-interacting proteins (RNA-independent) For each interacting protein, the CBC subunit mediating the interaction, the species in which the interaction was detected and the function of the interaction are presented. SLBP, stem–loop-binding protein; ZC3H18, zinc finger CCCH-type containing 18.

Interacting protein	CBC subunit	Species detected	Function	Reference(s)
Importin-α	Cbp80	*H. sapiens*, *S. cerevisiae* and *X. laevis*	Translocating CBC to nucleus, regulating the release of cargo from CBC and regulating CBC activity	[114,119,160]
Cdk9/Bur1p	Unclear	*H. sapiens* and *S. cerevisiae*	Transcription elongation and splicing	[63,64]
U4/U6.U5 tri-snRNP	Unclear	*H. sapiens*	Splicing	[34]
NELF-E	Unclear	*H. sapiens*	Histone RNA 3′ end processing	[32]
SLBP	Unclear	*H. sapiens*	Histone RNA 3′ end processing	[32]
Ars2	Unclear	*H. sapiens* and *D. melanogaster*	Histone RNA 3′ end processing and miRNA biogenesis	[98,102,103]
PARN	Cbp80	*H. sapiens*	mRNA stability	[108]
ZC3H18	Unclear	*H. sapiens*	Unclear	[34,48]
PHAX	Unclear	Mammalian cells and *X. laevis*	U snRNA nuclear export	[115,116]
Aly component of TREX	Cbp80	*H. sapiens*	mRNA nuclear export	[121,125]
hnRNP C	Cbp80	*H. sapiens*	mRNA nuclear export	[126]
UPF1	Cbp80	Mammalian cells	NMD	[150,153]
eIF4G	Cbp80	Mammalian cells and *S. cerevisiae*	CBC-dependent translation	[128,134,135]
CTIF	Cbp80	Mammalian cells	CBC-dependent translation	[136,137]

eIF4E is the cap-binding subunit of eIF4F, a complex required for cap-dependent translation initiation [24]. In the eIF4F complex, eIF4E binds to eIF4G, a scaffold protein to which other factors are recruited, including eIF4A, a DEAD-box RNA helicase required for 5′-UTR unwinding and eIF4G. The interaction of eIF4G with eIF4E is required for efficient 7mG binding [25,26]. Since there are many excellent reviews discussing eIF4F function [3,27,28], the present review will focus on the function and regulation of CBC. CBC is a multifaceted complex essential for gene expression, which integrates RNA processing events, transcript nuclear export and translation.

## IDENTIFICATION OF CBC AS A NUCLEAR CAP-BINDING COMPLEX

CBC was first purified from nuclear extracts of HeLa cells on the basis of its affinity for 7mG [29,30]. CBC was demonstrated to consist of 20 and 80 kDa polypeptides, which were designated as Cbp20 (cap-binding protein 20) and Cbp80 respectively. It is likely that CBC is present in all eukaryotes, and its evolution correlates with the appearance of 7mG. Cbp20 is unlikely to be present in significant quantities as a monomer since it is unstable in the absence of Cbp80, both in mammals and yeast [31–34], and is undetectable in Cbp80-immunodepleted extracts [29,35]. Conversely, it is not clear whether Cbp80 can exist as a monomer and Cbp20 is not required for Cbp80 stability [33].

Cbp20 and Cbp80 bind to 7mG synergistically and neither subunit alone has significant affinity for the structure [29,30,36]. The crystal structure of CBC revealed that the 7mG-binding pocket resides in Cbp20, and this was validated by mutational analysis [37]. On binding to CBC, 7mG is positioned between two conserved tyrosine residues (Tyr^20^ and Tyr^43^) in the Cbp20 subunit [38,39], and biophysical studies indicated that these residues are essential for the interaction [38,40]. Cbp80 causes a conformational change in Cbp20, which is required for CBC to bind to 7mG with high affinity. The Cbp80 structure is highly ordered and composed of three helical domains connected by two linkers. The Cbp80 N-terminal helical domain is structurally similar to MIF4G (middle domain of eIF4G), and is required for cap-dependent translation [37,38,41]. The MIF4G and intermediate helical domains of Cbp80 mediate interaction with Cbp20.

In addition to binding 7mG, CBC binds directly to RNA via both subunits. Cbp20 contains a classical RRM (RNA-recognition motif). A splice variant of Cbp20 that does not bind to Cbp80 or 7mG, but does contain a portion of the RRM, retains RNA-binding activity, albeit reduced [42]. Cbp80 also binds to RNA [43,44]. As described throughout the present review, CBC often has gene-specific effects. It is possible that the RNA-binding domains of CBC may have an enhanced affinity for specific RNA sequences or motifs and thus have a role in mediating these gene-specific effects.

CBC interacts with transcripts shortly after transcription in the nucleus. Although one of its functions is to accompany the transcript through the nuclear pore (described in detail later), it is a predominantly nuclear complex. Cbp80 contains a nuclear localization signal at the N-terminus, which is required for its nuclear localization [45,46], and Cbp20 is likely to be co-imported into the nucleus with Cbp80 [47].

CBC recruits several factors to 7mG-modified transcripts which mediate processing events [34,48,49]. The contribution of CBC to gene expression has been addressed in yeast, plants and mammalian cells by depleting and reducing the expression of the subunits. *CBP80* or *CBP20* deletion in *Saccharomyces cerevisiae* results in significant changes in gene expression, with many genes exhibiting a change of 2-fold or more [50,51]. Although in *S. cerevisiae CBP80* and *CBP20* are not essential for cell viability [31,52,53], they are required for cell growth and proliferation [31,54]. Disruption of CBC genes in *Arabidopsis thaliana* is not lethal, but results in developmental delays, reduced stature and ABA (abscisic acid) hypersensitivity owing to a down-regulation of transcripts involved in ABA signalling [55,56]. In mammalian cells, siRNA-mediated depletion of Cbp80 results in deregulation of approximately 400 genes and a significant reduction in the cell proliferation rate [32]. To our knowledge there are no reports of Cbp80 or Cbp20 gene deletion in mammalian systems, and therefore it is not clear whether CBC is required for embryonic development or mammalian cell viability.

## CBC AND TRANSCRIPTION

7mG formation is a co-transcriptional process and CBC is rapidly recruited to this structure during transcript synthesis. Using ChIP assays, Cbp20 and Cbp80 subunits were detected at the 5′ end of genes as well as within the gene bodies, suggesting that CBC may track with the elongating polymerase [32,57–61]. CBC is likely to be recruited to chromatin via an interaction with 7mG since monomeric Cbp20 and Cbp80 fail to be recruited [33,60], recruitment does not occur in Abd1p (cap methyltransferase)-deficient *S. cerevisiae* [33], and recruitment is mediated by RNA [62].

CBC can have a reciprocal relationship with transcription. The complex directly and indirectly recruits several transcriptional factors to promoters and for a subset of genes has an active role in transcription regulation [33,60,61,63,64]. In *S. cerevisiae*, CBC directly recruits Mot1p to a subset of gene promoters. Mot1p is a regulator of transcription that positively or negatively regulates the expression of RNA pol II-transcribed genes in a gene-specific manner [60]. On genes that Mot1p activates, CBC-dependent Mot1p recruitment results in the recruitment of general transcription factors and RNA pol II to promoters, stimulating transcription initiation. On genes that Mot1p represses, CBC-dependent Mot1p recruitment results in repression of RNA pol II recruitment [60]. It is currently unclear whether the mammalian homologue of Mot1p, BTAF1, has similar CBC-dependent functions. This CBC function is less likely to be prominent in mammals, since in these species the rate-limiting step in transcription, in general, is not recruitment of RNA pol II, but rather escape of promoter-proximal paused RNA pol II. CBC also indirectly interacts with Npl3p, an hnRNP (heterogeneous nuclear ribonucleoprotein)-like protein essential for growth in yeast [65]. CBC and Npl3p act synergistically to suppress transcription termination at weak polyadenylation sites by repressing the recruitment of the termination complex CF IA (cleavage factor IA) [33]. CBC also recruits Bur1p and Ctk1p, kinases that phosphorylate RNA pol II CTD and transcription factors, promoting transcriptional elongation [66,67]. CBC-dependent recruitment of Bur1p and Ctk1p stimulates RNA pol II CTD Ser^2^ phosphorylation and results in recruitment of histone methyltransferases and induction of H3K36me3 (histone H3 trimethylated on Lys^36^), a mark of active transcription [61,64].

**Figure 4:**
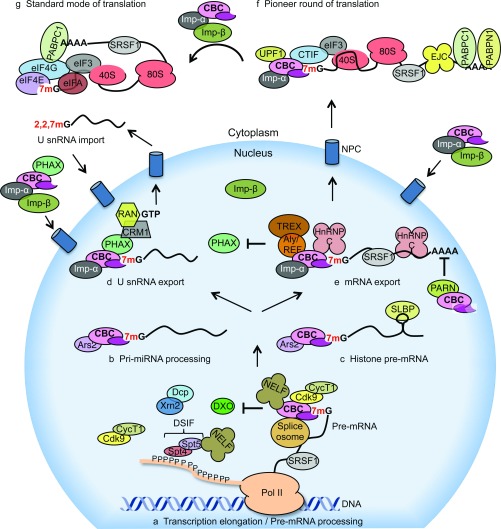
CBC functions in eukaryotic gene expression (a) CBC is required for pre-mRNA processing. The co-transcriptional binding of CBC to 7mG prevents the decapping activities of pre-mRNA degradation complexes [DXO (decapping exoribonuclease) and Dcp (decapping mRNA) Xrn2 (5′–3′ exoribonuclease 2)] and promotes pre-mRNA processing. CBC recruits P-TEFb [Cdk9/Cyclin T1 (CycT1)] to transcription initiation sites of specific genes promoting phosphorylation of the RNA pol II CTD at Ser^2^ residues. This results in the recruitment of splicing factors including SRSF1, which regulates both constitutive and alternative splicing events. Furthermore, CBC interacts with splicing machinery components that results in the spliceosomal assembly. CBC interacts with NELF and promotes pre-mRNA processing of replication-dependent histone transcripts. (b) CBC forms a complex with Ars2 and promotes miRNA biogenesis by mediating pri-miRNA processing. (c) CBC/Ars2 promotes pre-mRNA processing of replication-dependent histone transcripts. (d) CBC promotes export of U snRNA. CBC interacts with PHAX, which recruits export factors including CRM1 and RAN·GTP. (e) CBC promotes export of mRNA. For export of transcripts over 300 nucleotides, hnRNP C interacts with CBC and inhibits the interaction between CBC and PHAX, allowing the CBC to interact with TREX and the transcript to be translocated to the cytoplasm. CBC interacts with the PARN deadenylase and inhibits its activity, protecting mRNAs from degradation. (f) CBC mediates the pioneer round of translation. Cbp80 interacts with CTIF, which recruits the 40S ribosomal subunit via eIF3 to the 5′ end of the mRNA for translation initiation. Upon binding of importin-β (Imp-β) to importin-α (Imp-α), mRNA is released from CBC and binds to eIF4E for the initiation of the standard mode of translation. CBC-bound mRNP components not found in eIF4E-bound mRNPs are CTIF, exon junction complex (EJC) and PABPN1. (g) The standard mode of translation is mediated by eIF4E cap-binding protein. eIF4E is a component of the eIF4F complex which promotes translation initiation.

In mammals, CBC also stimulates transcription elongation of a subset of genes via the recruitment of P-TEFb (positive transcription elongation factor b), which contains Cdk9 (cyclin-dependent kinase 9; the mammalian homologue of Bur1p), to promoter-proximal paused RNA pol II (Figure 4) [63]. Depletion of CBC results in decreased RNA pol II CTD Ser^2^ phosphorylation, accompanied by reduced RNA pol II in the body of a subset of genes [63]. It is currently unclear whether CBC influences transcription of a subset of genes or acts genome- wide.

## CBC AND PRE-mRNA SPLICING

Eukaryotic pre-mRNA transcripts are synthesized as precursors containing an alternating series of exons and introns. The process of splicing excises introns and joins exons together to form the mature transcript [68]. The molecular machinery that catalyses splicing is called the spliceosome and is composed of five small nuclear ribonucleoprotein particles (U snRNPs) associated with a large number of additional proteins [69,70]. 7mG and CBC are required for efficient pre-mRNA splicing.

Incubation of uncapped or 7mG-capped transcripts with total or nuclear HeLa cell extracts initially revealed that the 7mG moiety is required for efficient splicing in mammalian systems [29,71–74]. Depletion of CBC from HeLa cell extracts resulted in inhibition of pre-mRNA splicing and reduced recruitment of U1 snRNP to the 5′ splice site of the 5′ proximal intron [29,35]. The effect of 7mG on splicing *in vivo* was initially observed in *Xenopus laevis* oocytes. Microinjected transcripts were only efficiently spliced if they were capped, and splicing of the 5′ proximal intron required 7mG [75]. Microinjection of *X. laevis* oocytes with antibodies raised against Cbp20 significantly decreased the splicing efficiency of microinjected transcripts [30].

In *S. cerevisiae*, only 3% of genes contain introns and are spliced [76]. Inactivation of the capping enzymes (Ceg1p or Abd1p) with temperature-sensitive mutants revealed that 7mG enhances splicing of a subset of these genes [77–80]. CBC is part of the splicing commitment complex [81], and depletion of CBC from cell extracts resulted in reduced *in vitro* splicing of pre-mRNA due to inhibition of spliceosome assembly [82,83]. The biological significance of the relationship between CBC and splicing is reflected in the observation that mutation of splicing components is synthetically lethal in CBC-deleted strains [31,84]. Furthermore, a Cbp20p mutant that cannot bind 7mG is synthetic lethal with the deletion of genes that encode for factors involved in the spliceosome assembly [85]. Notably, CBC has been demonstrated to couple splicing to transcription. Deletion of CBC results in a reduction in the recruitment of several splicing factors to the nascent transcript resulting in inhibition of co-transcriptional spliceosome assembly [86]. The dependency of splicing on CBC has also been observed in *A. thaliana* [87].

The mechanism by which CBC mediates splicing in mammalian cells has recently been investigated. A direct interaction of CBC with the protein components of the U4/U6.U5 tri-snRNP was observed, and these interactions were required for efficient co-transcriptional spliceosomal assembly [34]. Recent studies in mammalian cells have demonstrated that 7mG and CBC are required not only for the removal of 5′ proximal introns, but also for the removal of downstream introns [34,88]. It is currently unclear to what extent this mechanism is utilized throughout the genome.

Alternative splicing is the process by which the exons of a pre-mRNA are selected in different arrangements, producing multiple mRNAs, some of which produce distinct protein variants [89]. Previously, CBC was demonstrated to regulate alternative splicing in mammalian cells and in *A. thaliana* [63,90]. The model proposed is that CBC acts as a platform for the recruitment of splicing factors such as SRSF1 (serine/arginine-rich splicing factor 1) to elongating RNA pol II [63]. SRSF1 regulates constitutive and alternative splicing and has also been demonstrated to regulate cap-dependent translation initiation in the cytoplasm [91] (Figure 4).

## CBC AND PRE-mRNA 3′ END PROCESSING

Most mammalian mRNAs have a poly(A)-tail consisting of 200–250 adenosines at the 3′ end which protects transcripts from degradation and stimulates their translation. Formation of the poly(A)-tail or polyadenylation is a two-step process. Initially the pre-mRNA is cleaved at the poly(A) site followed by polyadenylation of the 3′ end [5]. *In vitro* studies demonstrated that m7G-capped pre-mRNAs were cleaved more efficiently at the poly(A) sites than uncapped pre-mRNA [92,93], and the same observation was made with transcripts microinjected into *X. laevis* oocytes [94]. In mammalian cells, m7G-capped transcripts were demonstrated to be more efficiently cleaved at the poly(A) site than incompletely capped or uncapped transcripts [88,95]. This effect was mediated by CBC [96]. Although the exact molecular mechanism by which CBC promotes pre-mRNA cleavage at the poly(A)-site remains to be determined, it is likely to involve interactions of CBC with the polyadenylation machinery [96].

CBC also has a role in other 3′-end processing events. Replication-dependent histone mRNAs, unlike most other mRNAs, do not possess a poly(A)-tail, but have instead a conserved 3′ end stem–loop structure [97]. In mammalian cells, depletion of CBC causes aberrant production of poly(A)-tailed histone mRNAs [32,98]. CBC interactions with NELF (negative elongation factor) and Ars2 (arsenite-resistance protein 2) are required for efficient histone mRNA 3′-end processing (Figure 4) [32,98]. Recent evidence suggests that CBC can also determine the fate of replication-dependent histone mRNAs towards degradation or translation [99].

The effect of CBC on 3′-end processing is species-specific. In *S. cerevisiae*, 7mG appears to have minimal effect on pre-mRNA 3′-end processing. Inactivation of the RNA guanylyltransferase Ceg1p exhibits no overt effects on polyadenylation levels [77]. This suggests that the effect on mRNA 3′-end formation is a CBC function that evolved later [47]. However, as described above, CBC can suppress aberrant 3′-end processing in *S. cerevisiae.* CBC acts in synergy with Npl3p to repress the recruitment of the termination complex CF IA to weak polyadenylation sites [33].

## CBC AND miRNA BIOGENESIS

miRNAs are endogenous transcripts that post-transcriptionally regulate gene expression in animals and plants. They are transcribed by RNA pol II as pri-miRNAs, thus carry 7mG and the poly(A)-tail [100]. During nuclear and cytoplasmic processing events, the pri-miRNA loses the 7mG and the poly(A)-tail and the mature, 21–23 nucleotide-long, miRNA is incorporated into RISC (RNA-induced silencing complex) to guide RNA silencing [101]. Ars2 and CBC have been demonstrated to be required for the biogenesis of a subset of miRNAs (Figure 4) [98,102,103]. Ars2 forms a complex with CBC and 7mG in mammalian cells [102]. Depletion of Ars2 with Cbp20 and Cbp80 from mammalian cells resulted in a decrease in miRNA biogenesis in a transcript-specific manner [98,102]. The same phenomenon was also observed in *Drosophila melanogaster* and *A. thaliana* [87,103,104].

## CBC AND RNA STABILITY

Removal of 7mG by decapping enzymes leads to RNA degradation. This process can be regulated in a gene-specific manner by signalling pathways resulting in a biological response [105]. Electroporation of *in vitro* transcribed RNAs containing 7mG or incompletely capped structures into mammalian cells revealed that 7mG is required to stabilize mRNA [106]. The stability is, in part, mediated by cap-binding proteins, such as eIF4E, PABPC1 and CBC, that compete with the decapping enzymes for the 7mG structure [21,88,106,107]. Furthermore, CBC also interacts with and inhibits PARN deadenylase, which catalyses poly(A)-tail removal, an initial step in mRNA degradation (Figure 4) [108].

Recently, decapping and transcript degradation was demonstrated to occur co-transcriptionally and to limit bidirectional RNA pol II elongation and the production of aberrantly processed pre-mRNAs [88,109,110]. This suggests that the competition between CBC and the decapping complexes could regulate the balance between transcription elongation and degradation of the nascent transcript (Figure 4). CBC was also identified to interact with the NEXT (nuclear exosome targeting) complex, although the biological significance of this interaction remains unclear [34,111].

## CBC AND RNA POL II TRANSCRIPT EXPORT: U snRNAs

In eukaryotic cells, RNA pol II transcripts are synthesized in the nucleus, but undergo essential processing and/or translation in the cytoplasm. Therefore transcript nuclear export is a key step in gene expression. U snRNAs, although synthesized and functioning in the nucleus, are processed and receive their protein partners in the cytoplasm. Efficient nuclear export of U snRNAs was found to be 7mG-dependent in *X. laevis* oocytes and requires CBC [30,112–114]. In order for CBC to promote U snRNA nuclear export, it interacts with PHAX (phosphorylated adaptor of RNA export) [115,116]. Upon phosphorylation by CK2 kinase, PHAX stimulates the nuclear export of U snRNAs in a process mediated by CRM1 in a RAN·GTP-dependent manner [115,117]. In the cytosol, PHAX dephosphorylation and importin-β are required for U snRNA release and recycling of CBC/PHAX back to the nucleus [115,117].

In *S. cerevisiae*, 30% of importin-α is isolated in a complex with CBC [114]. This interaction has also been observed in *X. laevis* oocytes and mammalian cells [114,118], and validated by co-crystallization of CBC with importin-α [119]. In the nucleus, importin-α/CBC binds to 7mG–U snRNA and the complex is exported. In the cytosol, importin-β interacts with importin-α and releases the U snRNA for processing including the formation of the 5′ TMG (2,2,7-trimethylguanosine cap) [114,119]. Subsequently, the importin-α/importin-β/CBC complex is re-imported into the nucleus. High levels of RAN·GTP in the nucleus promote dissociation of importin-β from the complex, and a new cycle begins (Figure 4). Of note, after nuclear re-entry of U snRNAs, the presence of the TMG prevents efficient binding by CBC [120].

## CBC AND RNA POL II TRANSCRIPT EXPORT: mRNA

In higher eukaryotes, CBC stimulates mRNA nuclear export via an interaction with Aly/REF [121,122]. Aly/REF is a component of TREX (transcription export complex), a multi-subunit protein complex that links pre-mRNA processing with mRNA nuclear export in mammalian cells, required for the export of spliced and unspliced mRNAs [123]. TREX interacts with TAP, the mRNA export receptor [124]. Thus CBC promotes mRNA nuclear export by facilitating the recruitment of nuclear export machinery to the transcript (Figure 4) [121,122,125].

As described above, U snRNA and mRNA are exported by distinct mechanisms. The factor that distinguishes between these mechanisms is transcript length. The default export pathway utilizes PHAX. However, when CBC is bound to transcripts longer than 300 nucleotides, it interacts with hnRNP C, which abolishes the interaction between CBC and PHAX, thus selecting the TREX export pathway (Figure 4) [126].

In *S. cerevisiae*, both CBC and 7mG are dispensable for the majority if not all mRNA export [77,127].

## CBC AND THE PIONEER ROUND OF TRANSLATION

The majority of translation in eukaryotes is dependent on eIF4E binding to 7mG, which drives translation initiation by recruiting the 40S ribosomal subunit [3,24]. CBC has an analogous function to eIF4F in translation with the exception that it drives the first round or rounds of translation known as the pioneer round of translation. The pioneer round of translation was originally identified by a screen performed in *S. cerevisiae* in which a Cbp80p mutant was found to be synthetic lethal with an eIF4G mutant deficient for eIF4E and Pab1p binding [128]. Although the pioneer round of translation was initially believed only to have a quality control role in orchestrating NMD (nonsense-mediated decay) [129,130], evidence suggests that CBC-bound transcripts can undergo multiple rounds of translation generating functional products [131–133]. However, in contrast to the standard mode of translation mediated by eIF4F, the pioneer round of translation does not generate abundant amounts of protein.

The mechanism of CBC-mediated translation initiation is not well characterized and is somewhat controversial. CBC has been demonstrated to interact with eIF4G in yeast and mammalian cells [128,134,135]. However, a novel protein with similarity to eIF4G designated as CTIF (CBC-dependent translation initiation factor) was demonstrated to interact with Cbp80 [136]. CTIF is required during the initiation of the pioneer round of translation, but is not involved in the standard mode of translation [136,137]. CTIF interacts with eIF3g, a component of the eIF3 complex, and recruits the 40S ribosomal subunit to CBC-bound mRNA for the pioneer round of translation (Figure 4) [137]. Depletion of CTIF causes redistribution of CBC from polysomes to subpolysomal fractions, suggesting that CTIF is an essential component of the pioneer round of translation that functions in a manner similar to eIF4G in the standard mode of translation [136,137]. In contrast with the eIF4F-dependent translation where the eIF4E-bound mRNA forms a circular structure [138,139], similar circularization has not been observed in CBC-dependent translation [140].

The transition from the pioneer round of translation to the standard mode of translation and the exchange of CBC for eIF4F at 7mG is regulated by importins [118]. Importin-α, as described previously, interacts with Cbp80 [114,119]. In the cytosol, importin-β interacts with importin-α and promotes the dissociation of the mRNA from CBC. Subsequently, eIF4E interacts with 7mG promoting eIF4E-dependent translation initiation (Figure 4) [118].

Fundamental questions remain concerning the mechanism and biological significance of the pioneer round of translation. It is currently unclear what proportion of transcripts utilize the pioneer round of translation, to what extent and under which growth conditions.

## CBC AND NONSENSE-MEDIATED DECAY

NMD is a mRNA surveillance pathway that predisposes aberrant mRNAs containing a PTC (premature termination codon) for degradation [141]. NMD can play an important role in regulating the steady-state levels of a subset of mRNAs, modulating several biological responses [142]. The major facilitators of NMD are the UPF proteins (UPF1, UPF2 and UPF3), which are required to detect PTCs [143].

NMD is a translation-dependent process and transcripts engaged in CBC-, eIF4E- or IRES (internal ribosome entry site)-mediated translation are all subject to NMD [140,144–149]. UPF1 can be co-purified with CBC- and eIF4E-bound transcripts in an RNA-dependent manner [140,144,145,149]. Therefore NMD can act at any round of translation and is independent of a specific translation initiation mechanism [146,148,149].

In mammalian cells, siRNA-mediated depletion of Cbp80 results in partial inhibition of NMD [136,150]. However, in *S. cerevisiae* and *A. thaliana* CBC is dispensable for NMD [147,151,152]. In mammalian cells, CBC was demonstrated to interact physically with UPF1 via Cbp80 (Figure 4) [150,153], and inhibition of this interaction abolished NMD [153]. Although the exact molecular details are unclear, CBC interaction with UPF1 promotes NMD machinery assembly on the aberrant mRNA, promoting decay [150,153]. However, there is controversy regarding the direct interaction between CBC and UPF1 since several studies have failed to detect it after RNase treatment [140,154].

## REGULATION OF CBC

Gene expression is a heavily regulated process and examples are emerging of the regulation of CBC function. Growth factors and mTORC1 (mammalian target of rapamycin complex 1) kinase have been demonstrated to regulate CBC affinity for 7mG [119,155–157]. Nutrient availability and growth factors activate mTORC1 kinase, which phosphorylates S6 kinase [158]. S6 kinase phosphorylates Cbp80, stimulating the affinity of CBC for 7mG *in vitro* [156]. *In vivo*, Cbp80 phosphorylation was observed to be stimulated by growth factors and could be inhibited by rapamycin, an mTORC1 inhibitor. Increased Cbp80 phosphorylation was observed to correlate with increased cap-dependent splicing activity [156], and the recruitment of the S6 kinase to CBC-bound mRNPs (messenger ribonucleoproteins) stimulated translation efficiency [159]. Interestingly, binding of importin-α and importin-β to CBC was essential for growth-factor-mediated stimulation of 7mG binding [119]. Furthermore, overexpression of constitutively active RAN within cells, which abolishes the interaction between importin-β and the CBC–importin-α complex, stimulates CBC cap-binding activity [160].

## SPECIES-SPECIFIC EFFECTS OF CBC

As discussed throughout the present review, certain CBC functions are observed in all species that express the complex, whereas other functions have only been observed in a subset of species. During evolution, selective pressure has resulted in divergence of gene expression mechanisms, including those involving CBC [161]. Cbp20 is primarily responsible for 7mG binding and is highly conserved. However, Cbp80 is a platform for recruiting proteins to the transcript and appears to have evolved to co-ordinate the increasing complexity of gene expression mechanisms in mammals [37,39,114]. CBC binds to NELF-E, Ars2 and CTIF to mediate histone pre-mRNA processing, miRNA biogenesis and the pioneer round of translation respectively [32,98,102,103,136]. Homologues of NELF-E, Ars2 and CTIF have not been identified in yeast and, to our knowledge, in *S. cerevisae* these events are not mediated by CBC [50,77].

Splicing is another gene regulation mechanism which has changed dramatically during evolution. In *S. cerevisiae*, for example, only 3% of genes contain introns and therefore splicing is not prevalent, whereas conversely the majority of metazoan genes contain multiple introns [76]. In metazoans, several gene expression steps have evolved to become coupled to splicing which is obviously not the case in *S. cerevisiae* [162]. This may explain why CBC is dispensable for mRNA export and NMD in *S. cerevisiae*, since it interacts with the splicing machinery during these processes.

However, when discussing species specificity it must be stressed that most information about CBC function (and all gene expression mechanisms) comes from a limited number of organisms, and therefore the true extent of conservation of CBC function is not clear. Furthermore, different model organisms are amenable to distinct forms of experimentation, which may result in the appearance of differences in CBC function. For example, in *S. cerevisiae*, temperature-sensitive mutants allow inhibition of protein function and/or expression within minutes, whereas in human cells siRNA-mediated suppression of CBC leads to loss of protein expression over hours and days, which may lead to indirect effects being observed. Research into the function of CBC may benefit from an inhibitor of the Cbp20–Cbp80 interaction, which would rapidly inhibit CBC function, thus reducing the observation of indirect effects.

## FUTURE PERSPECTIVES

There has been intense research into CBC function over the last decade, which has raised many intriguing questions about how this complex influences gene expression.

Do Cbp20 and Cbp80 have CBC-independent functions? The majority of Cbp20 and Cbp80 functions are as a heterodimer. However, there is evidence that these subunits may also function independently. Cbp80, but not Cbp20, was co-purified with eIF4E-bound transcripts, indicating that a pool of Cbp80 exists independently of Cbp20 [140]. Furthermore, gene expression profiling of *CBP20*- and *CBP80*-deletion strains in *S. cerevisiae* revealed that only 15% of regulated transcripts were common to both strains, suggesting that these subunits have independent functions [50]. The question of whether Cbp80 has an independent role is experimentally important since the function of CBC is often inferred from using reagents that only target Cbp80 expression or interactions. An alternatively spliced isoform of Cbp20, Cbp20s, was identified which is likely to function independently of Cbp80, since it does not interact with either Cbp80 or 7mG [42]. The novel Cbp20 isoform was observed to bind to mRNA, presumably via a conserved RRM domain, and was recruited to active transcription sites. It will be interesting to determine the Cbp80 and m7G-independent functions of Cbp20s, and whether Cbp20 also has these functions in the CBC complex.

Does CBC have enhanced affinity for certain transcripts? Many of the studies described in the present review have provided evidence that CBC exhibits transcript specificity. Both Cbp20 and Cbp80 can bind to transcripts independently of 7mG and therefore it is plausible that these subunits could add specificity to the interaction with RNA [42–44].

What is the role of CBC in long non-coding RNA processing? Over recent years there has been explosion of knowledge and interest in lncRNAs (long non-coding RNAs) and other pervasive 7mG-containing transcripts [163,164]. It seems likely that CBC will bind to these transcripts and therefore an obvious question is whether CBC has effects on their metabolism and function.

Can CBC be targeted therapeutically? Although there is no direct evidence to suggest that Cbp20 or Cbp80 are proto-oncogenes, mRNA expression profiling has detected elevated expression of Cbp20 in a subset of tumours [165]. Moreover, as described previously, CBC function is regulated by the mTOR signalling pathway, which when deregulated causes cell transformation [166,167]. Considering that a common feature of tumour cells is enhanced gene expression rates, tumour cells may be predicted to be more sensitive than untransformed cells to inhibition of CBC function. Inhibitors of the Cbp20–Cbp80 or Cbp20–7mG interactions would be not only useful experimentally to determine the direct functions of CBC, but could also be used to determine whether this interface should be pursued as a therapeutic target.

## CONCLUSION

CBC plays a pivotal role in post-transcriptional processing events. CBC binds to RNA pol II transcripts during transcription and accompanies them through much of their lifetime, facilitating processing events, including transcription regulation, pre-mRNA processing, mRNA export and the pioneer round of translation (Table 1 and Figure 3). With the first discoveries of regulation of CBC function, it is an exciting possibility that CBC is a complex through which cellular signalling pathways can change the gene expression landscape and cellular physiology.
